# Elongation and Contraction of Scallop Sarcoplasmic Reticulum (SR): ATP Stabilizes Ca^2+^-ATPase Crystalline Array Elongation of SR Vesicles

**DOI:** 10.3390/ijms23063311

**Published:** 2022-03-18

**Authors:** Jun Nakamura, Yuusuke Maruyama, Genichi Tajima, Makiko Suwa, Chikara Sato

**Affiliations:** 1 Health and Medical Institute, National Institute of Advanced Industrial Science and Technology (AIST), Central 6, 1-1-4 Umezono, Tsukuba 305-8568, Ibaraki, Japan; yuusuke.maruyama@gmail.com; 2 Institute for Excellence in Higher Education, Tohoku University, 41 Kawauchi, Aoba-ku, Sendai 980-8576, Miyagi, Japan; g_tajima@tohoku.ac.jp; 3 Biological Science Course, Graduate School of Science and Engineering, Aoyama Gakuin University, 5-10-1 Fuchinobe, Chuou-ku, Sagamihara 252-5258, Kanagawa, Japan; suwa@chem.aoyama.ac.jp

**Keywords:** scallop, sarcoplasmic reticulum, Ca^2+^-ATPase, two-dimensional crystallization, ATP, membrane endoskeleton, transmission microscopy, cell morphology, cell dynamics, thapsigargin

## Abstract

The Ca^2+^-ATPase is an integral transmembrane Ca^2+^ pump of the sarcoplasmic reticulum (SR). Crystallization of the cytoplasmic surface ATPase molecules of isolated scallop SR vesicles was studied at various calcium concentrations by negative stain electron microscopy. In the absence of ATP, round SR vesicles displaying an assembly of small crystalline patches of ATPase molecules were observed at 18 µM [Ca^2+^]. These partly transformed into tightly elongated vesicles containing ATPase crystalline arrays at low [Ca^2+^] (≤1.3 µM). The arrays were classified as ‘’tetramer’’, “two-rail” (like a railroad) and ‘’monomer’’. Their crystallinity was low, and they were unstable. In the presence of ATP (5 mM) at a low [Ca^2+^] of ~0.002 µM, “two-rail” arrays of high crystallinity appeared more frequently in the tightly elongated vesicles and the distinct tetramer arrays disappeared. During prolonged (~2.5 h) incubation, ATP was consumed and tetramer arrays reappeared. A specific ATPase inhibitor, thapsigargin, prevented both crystal formation and vesicle elongation in the presence of ATP. Together with the second part of this study, these data suggest that the ATPase forms tetramer units and longer tetramer crystalline arrays to elongate SR vesicles, and that the arrays transform into more stable “two-rail” forms in the presence of ATP at low [Ca^2+^].

## 1. Introduction

The sarcoplasmic reticulum (SR) is a type of endoplasmic reticulum specialized for the regulation of calcium concentration in muscle. The SR sequesters cytoplasmic Ca^2+^ released from the SR during muscle contraction; in this process integral transmembrane Ca^2+^-ATPases [1,2,3] transfer the Ca^2+^ ions into the SR lumen with the help of ATP. This lowers the cytoplasmic Ca^2+^ concentration, leading to the relaxation of the muscle [4,5]. Recently, SR vesicles isolated from the adult fast-twitch skeletal muscle of rabbit, were observed using negative staining and transmission electron microscopy (TEM) [6]. At a low Ca^2+^ concentration (≤0.9 nM), at which the SR Ca^2+^-ATPase molecules scarcely transport calcium, the surface ATPase molecules (40 Å diameter) formed crystalline arrays with the help of ATP to elongate the SR vesicles. As calcium concentration increased to a higher concentration (0.2 µM) at which the ATPase molecules fully perform their transport reaction, the ATPase crystalline arrays degraded. Based on these observations, we proposed that the ATPase molecules act as a calcium-sensitive, membrane endoskeleton of SR. However, in this study, the rabbit SR vesicles were too susceptible to cohesion/agglomeration in the presence of ATP to obtain the number of images required for statistical analysis, in particular, to measure the calcium dependencies of ATPase crystallization and vesicular morphology. Thus, the model of a calcium-sensitive membrane endoskeleton proposed for rabbit SR [6] remained preliminary. On the other hand, earlier the elongated SR vesicles isolated from the cross-striated adductor muscle of scallop were reported to have crystalline arrays of ATPase particles (~40 Å diameter), independent of ATP [7]. The sensitivity of the scallop crystals to Ca^2+^, however, was shown to be low and slow; 0.1 mM Ca^2+^-induced collapse of the crystals takes more than 15 min [8].

**Figure 1:**
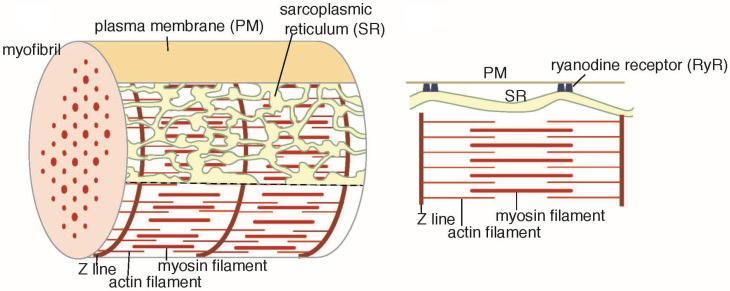
Diagram of a cross-striated adductor muscle cell of scallop in the resting state. The illustrations are based on the data of Castellani et al. (1989) [8], Sanger and Sanger (1985) [9], Loesser et al. (1992) [16] and Quinn et al. (1998) [17]. An illustration of the feet-like structure of the vertebrate cross-striated muscle can be found in [18].

The striated muscle of scallops differs from that of vertebrates as follows: (i) Each scallop muscle cell has only one myofibril at the center [9]; (ii) the SR of each scallop muscle cell forms a continuous tubular system in the zone beneath the cell surface, and it controls all of the sarcomeres; (iii) when the muscle is relaxed, the system is basically a branched tube of uniform diameter [8]; (iv) scallop SR directly associates with the cell surface membrane via junctional feet-like structures of ryanodine receptors (RyR: the calcium release channel of SR). These morphological features of the scallop are illustrated in Figure 1. In contrast, the SR of vertebrate muscle associates with the T-tubules via the junctional feet-like structures of RyRs [10,11,12]. ATPase of the scallop SR is adapted to cold; it has also been found to work at a low temperature, irrespective of its lipid environment [13].

A scallop SR preparation with a high Ca^2+^-ATPase percentage (~80% of the protein present) was employed in the earlier studies mentioned above, and its calcium transport activity (~4 nmol Ca^2+^/mg of protein/min) [7] was much lower than the high calcium transport (800–6000 nmol Ca^2+^/mg of protein/min) of rabbit skeletal muscle SR [14,15]. In the present work, we used the isolated scallop SR with a high calcium transport activity (~1000 nmol Ca^2+^/mg of protein/min) but with a low ATPase content [15], to find out whether the calcium dependence of crystalline array formation and elongation of the SR observed for rabbit ATPase proteins [6] is also true for scallop. In the first part of this study, the stability of the scallop ATPase crystalline array was examined in the presence and absence of ATP. During these experiments, we observed that the crystallization of scallop Ca^2+^-ATPase is differed from that of the rabbit SR; the crystalline arrays have structural units of the ATPase tetramers, and they were stabilized by ATP.

## 2. Results

### 2.1. Effect of ATP on Crystallization of ATPase Surface Particles of SR

**Figure 2:**
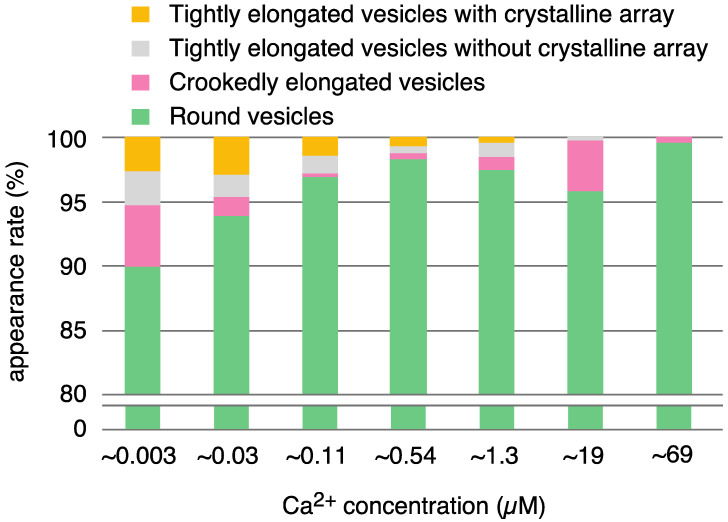
Overview showing the appearance rates of different types of SR vesicle relative to the total number of vesicles at ~0.003–69.0 µM Ca^2+^. The five-type classification of the vesicles shown in Table S1 was simplified to four types: tightly elongated vesicles with crystalline arrays (yellow), tightly elongated vesicles without a crystalline array, but including vesicles with an assembly of crystal patches or unclear arrays (gray), crookedly elongated vesicles (pink) and round vesicles (green). The crookedly elongated vesicles and round vesicles also sometimes had an assembly of crystal patches or unclear arrays. The rates employed are the average percentages (see Table S1) of the number of each vesicular type to the total number of vesicles at the respective calcium concentrations. For convenience, the rates axis has been truncated; the region below 80% is not completely shown for the round vesicles.

**Figure 3:**
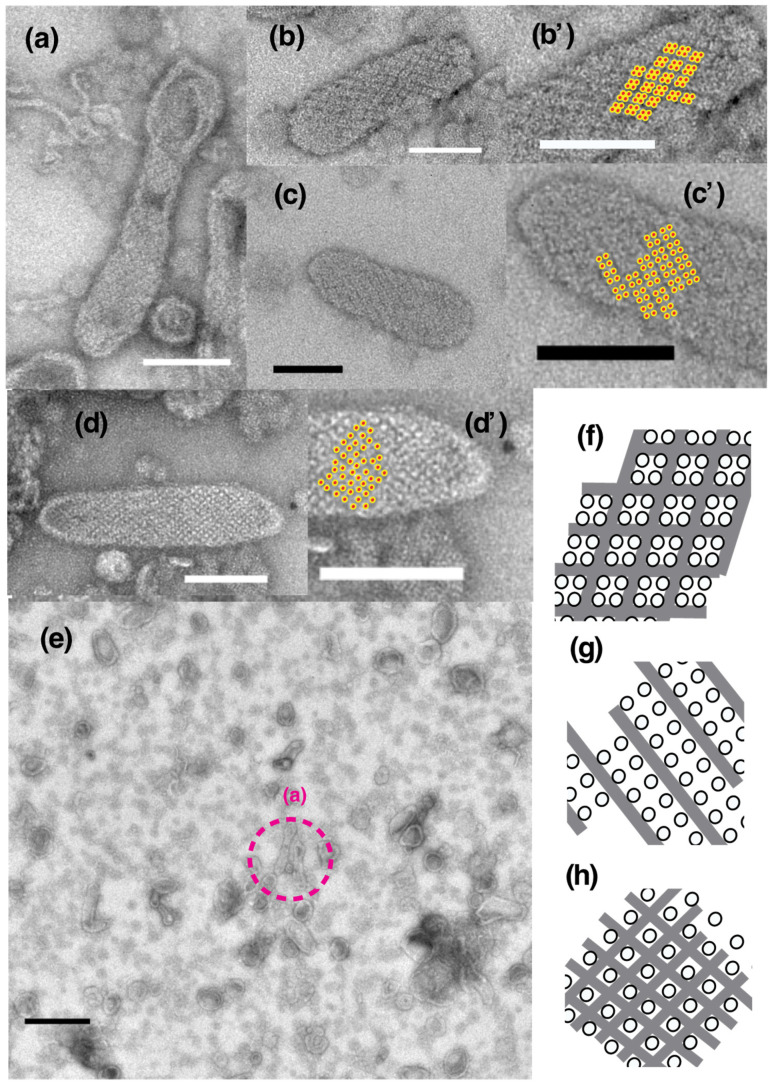
Typical images of tightly elongated SR vesicles, predominantly displaying one of the three different crystalline arrays: tetragonal, “two-rail” and monomer arrays of 40 Å particles. (**a**,**b**,**b’**) Vesicles with the tetragonal array. (**b’**) Higher magnification image of the vesicle in (**b**). The four particles within the tetragons observed in (**b**) are marked with the dotted circles in (**b’**) (see text for details). A darker trench was observed between the tetragonal units. (**c**) Vesicles with the “two-rail” array. (**c’**) Higher magnification image of the vesicle in (**c**). Each “two-rail” array was separated by a darker trench. (**d**) Vesicles with a monomer array. (**d’**) Higher magnification image of the vesicle in panel (**d**). Each monomer is surrounded by a darker trench. (**e**) An overview picture of the vesicle populations. The dotted-circle (**a**) shown in (**e**) indicates the vesicle with a tetragonal array shown in (**a**). (**f**–**h**) Illustrations of the tetragonal, two-rail and monomer arrays, respectively. The SR vesicle preparations were incubated at about 0.003 (**d**), 0.03 (**a**,**b**,**e**) and 0.11 (**c**) µM Ca^2+^ in the absence of ATP for 1 min after the addition of DDW (see “Section 4. Materials and Methods”). Scale bars in (**a**–**d**): 100 nm. Scale bar in (**e**): 0.5 µm.

Isolated scallop SR vesicles were observed at a range of calcium concentrations above 0.001 (0.003–69.0) µM in the absence of ATP to analyze the morphology of the SR vesicles and dispositions of the surface 40 Å ATPase particles under these conditions (Table 1 and Figure 2; see Supplementary Table S1 for further details). At 1.3 µM Ca^2+^ or less, crystalline arrays of ATPase appeared on some of the tightly elongated SR vesicles. Dispositions of ATPase in the crystals were classified as the follows: (i) Tetragonal: an array of tetragons (~100 Å square) (Figure 3a,b,b’) comprised of four ATPase particles; (ii) two-rail: a “two-rail” (like a railroad) array of monomers (Figure 3c,c’); (iii) monomer: an array of monomer particles (Figure 3d,d’). The classification was carried out manually by visual inspection. The three types of arrays are illustrated in Figure 3f–h, respectively. In tetragonal arrays, four particles can be clearly distinguished in some of the tetragons (Figure 3b’, the four particles in the tetragons are marked with dotted circles). The dimensions of each particle fit the molecular size of the top view of the ATPase protein (~40 Å diameter; e.g., see in [7]). Each tetragonal unit is surrounded by a darker trench (Figure 3b’). The surfaces of vesicles containing tetragonal arrays were predominantly covered by ATPase tetragons (see Figure 3a,b). In vesicles containing two-rail arrays, each “two-rail” was separated by darker trench lines (Figure 3c’). In vesicles containing monomer arrays, each monomer was surrounded by a darker trench (Figure 3d’). The size of the particles forming “two-rail” and monomer arrays also fits the molecular size of ATPase top views (Figure 3c,d).

**Figure 4:**
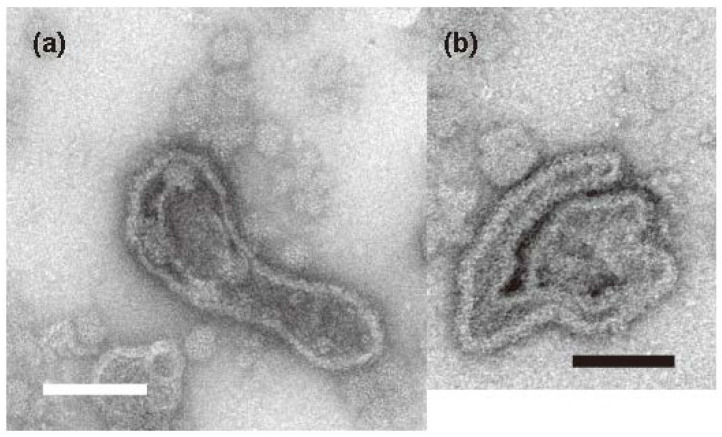
Typical images of crookedly elongated SR vesicles. SR vesicles were incubated at 0.002 (**a**) and 0.026 (**b**) µM Ca^2+^ in the absence of ATP for 1 min. Scale bar: 100 nm.

**Figure 5:**
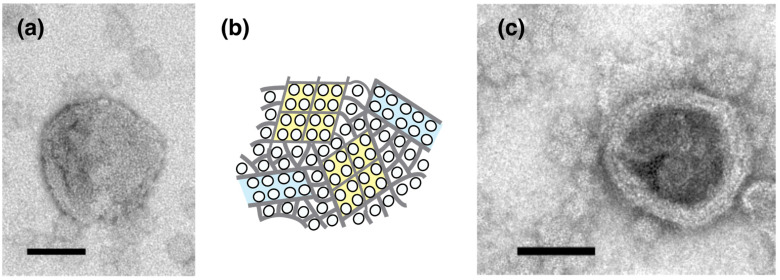
Typical images of the crystal patch assembly and unclear dispositions of the 40 Å particles. (**a**) vesicle containing crystal patch assembly. (**b**) Illustration of the crystal patch assembly. (**c**) Unclear disposition. SR vesicles were incubated at 0.026 (**a**) and 0.11 (**c**) μM Ca^2+^ in the absence of ATP for 1 min. Scale bar:100 nm.

Vesicles (major axis > 0.065 µm) within the preset areas (5.4 µm by 5.4 µm) were first classified as elongated or round depending on their axis ratio (see the legend of Table 1). Afterwards, the elongated vesicles were subclassified as tightly (see Figure 3a–d) or crookedly elongated (Figure 4a,b). Finally, depending on the disposition of their ATPase particles, the round, elongated and crookedly elongated vesicles were classified further into the categories: ‘crystalline arrays’ (see Figure 3a–d), ‘tight assembly of crystal patches’ (Figure 5a) and ‘unclear disposition’ (Figure 5c). The tight assemblies of crystal patches were composed of various types of 40 Å ATPase particle aggregate (e.g., fragmented ladder and “Lego” types) as illustrated in Figure 5b.

**Figure 6:**
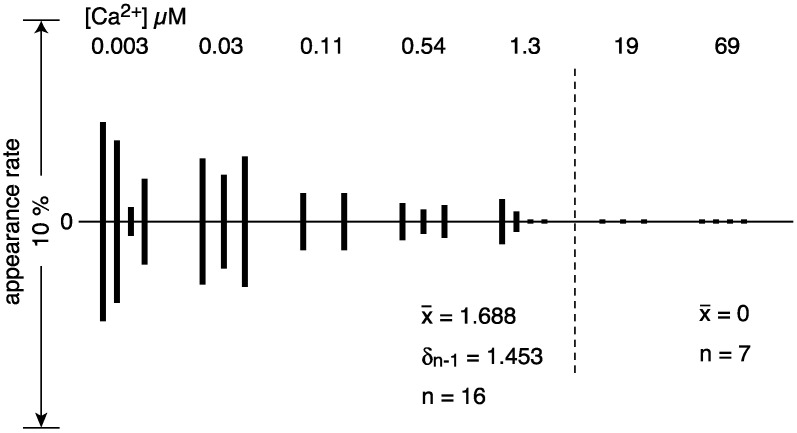
Percentage (%) of the number of tightly elongated vesicles with an ATPase crystalline array relative to the total number of vesicles in each of the four views recorded at the respective calcium concentration in the absence of ATP (see text for details). Vesicles with tetragon, two-rail or monomer crystals or a mixture of these crystal types, were included. Vesicles with crystalline arrays predominantly appeared at ~1.3 µM Ca^2+^ or less.

The number of vesicles containing crystals (i.e., vesicles with tetragon, two-rail or monomer crystals or their mixture) observed in each view was 1–7, which is only 0.7–4.9% of the total number of vesicles in the views recorded at the various calcium concentrations (ranging from ~0.003 to ~0.54 µM Ca^2+^). Moreover, the number of crystal-containing vesicles fluctuated a lot from view to view at the same calcium concentration (see Table S1 for details). Nevertheless, an attempt was made to find out whether changes in the shape of the tightly elongated vesicles and the disposition of ATPase particles were calcium-dependent or not. For example, percentages (%) (see Table S1) of the number of tightly elongated vesicles to the total number of vesicles at each Ca^2+^ concentration examined from ~0.003 to ~69 µM were displayed in an amplitude bar graph (Figure 6), to obtain a bird’s-eye view of the [Ca^2+^] dependence of ATPase molecule alignment. The amplitude graph (Figure 6) indicates that tightly elongated vesicles predominantly appear at ~1.3 µM Ca^2+^ or below (with an appearance rate of 1.68% (SD = 1.45, n = 16)), and completely disappear when the Ca^2+^ concentration becomes more than ~1.3 µM. Further, the percentage of crystal-containing tightly elongated vesicles (0.7–4.9%) present at a low calcium concentration (0.003–0.03 µM), tends to be higher than at calcium concentrations above 0.11 µM; more data are required for further discussion. We could not find conditions that resulted in a larger percentage of tightly elongated vesicles; up to now, tightly elongated vesicles were never the dominant species in the SR vesicle populations.

Tightly elongated vesicles containing crystal patch assemblies and unclear arrays also mainly appeared at ~1.3 µM Ca^2+^ or less and disappeared above ~1.3 µM [Ca^2+^] (Table 1, Figure 2 and Table S1), similar to the crystal-containing tightly elongated vesicles described above.

Crookedly elongated vesicles were (0–8 vesicles per view) observed at all of the calcium concentrations (0.003–69 µM) examined (Table 1, Figure 2 and Table S1). However, in contrast to the tightly elongated vesicles, no distinct crystalline arrays were observed. Thus, crookedly elongated vesicles are probably not only morphologically, but also qualitatively, different from tightly elongated vesicles.

**Figure 7:**
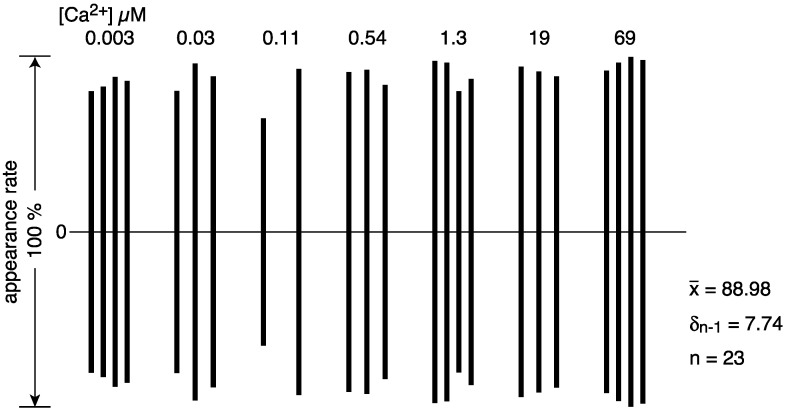
Percentages (%) of the number of round vesicles with a crystal patch assembly of ATPase relative to the total number of vesicles present in each of the four views recorded at the respective calcium concentration in the absence of ATP (see text for details).

The round vesicles present in the views employed did not have distinct large crystal-arrays of surface particles at any of the calcium concentrations investigated. In most cases, the particles displayed a tight assembly of crystal patches comprised of the different units (Figure 5a). Round vesicles with such crystal patch assemblies (88.98% (SD = 7.74, n = 23)) predominated over the other types of vesicle at every calcium concentration from ~0.003–~69.0 µM Ca^2+^ (Figure 7 (see Table S1 for details)).

The SR preparations used here were separated from the SR stock, which had a protein concentration of 6–13 mg/mL. Based on the amount of Ca^2+^ (16 ± 7 µM) present in the SR suspension containing 0.3 mg/mL protein (see “Section 4. Materials and Method”), the concentration of residual Ca^2+^ in the stock preparation is calculated to be larger than 180 µM. The calcium concentrations described below were adjusted with EGTA, taking into account of the total amount of calcium present in the solution, i.e., the supplemented and the residual calcium (see “Section 4. Materials and Methods” for details). This allowed the relationship between vesicular structure and Ca^2+^ concentration and lead to the following results: (i) The appearance rate of elongated vesicles containing crystalline arrays at ≤1.3 µM Ca^2+^ was low and only 1.68% (SD = 1.45, n = 16). In contrast, they disappeared completely at > 1.3 µM Ca^2+^ (Table 1, Figure 2 and Figure 6). (ii) The appearance rate of round vesicles with one or more crystal patch assemblies was high, over the calcium concentration range 0.003–69 µM, namely 88.98% (SD = 7.74, n = 23) (Table 1, Figure 2 and Figure 7). From this, the vesicles present in the stock at the high calcium concentration of 180 µM are deduced to have a round form and to contain crystal patch assemblies. These data suggest that round vesicles with a crystal patch assembly of ATPase in the SR stock could transform into tightly elongated vesicles with ATPase crystalline disposition as the calcium concentration (≤1.3 µM) decreases.

**Figure 8:**
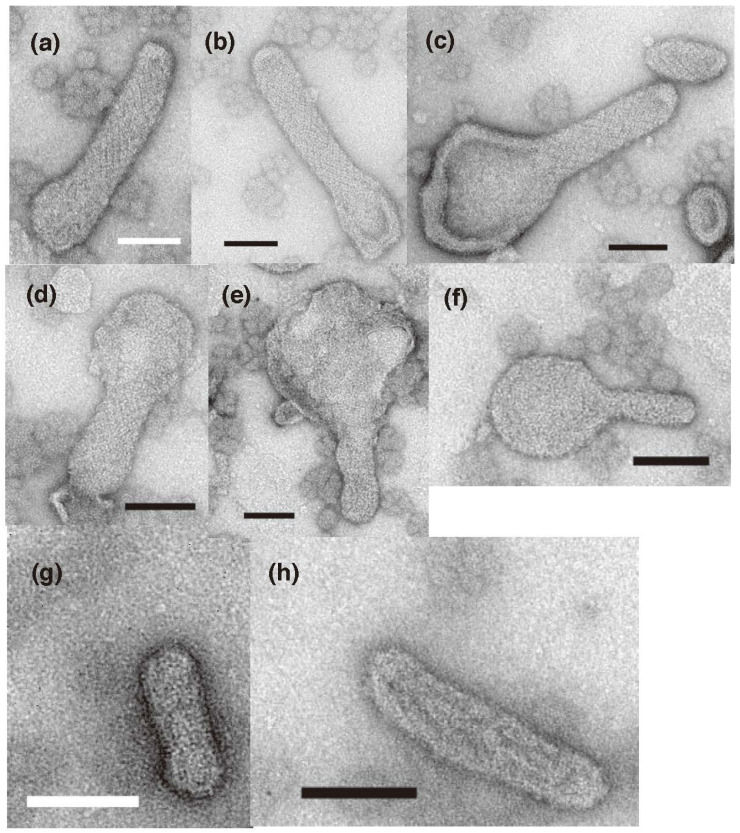
Time course of the morphology of SR vesicles during incubation at a low calcium concentration (~0.002 µM) in the absence of ATP. (**a**–**c**) SR vesicles after a 37-min incubation. (**d**–**f**) SR vesicles after 93 min. (**g**,**h**) SR vesicles after overnight incubation. Orderly arrays were degraded after 93 min and had disappeared after overnight incubation. The incubation was started by the addition of water. Before the addition of water, the SR preparation was incubated in the buffer solution for 5–9 min (see “Section 4. Materials and Methods”). Scale bar: 100 nm.

Earlier, it was reported that vesicles with “two-rail” ATPase crystalline arrays appear at less than 100 µM Ca^2+^, regardless of whether ATP is present or not [8]. The crystallinity of the two-rail arrays observed here was generally low (Figure 3c,c’), although the SR vesicles were pre-incubated at a low calcium concentration (<1.3 µM) for 1 min. It was assumed that this would favor their formation. The effect of prolonged incubation of the vesicles at a low calcium concentration (~0.002 µM) without ATP was therefore investigated. For this, SR preparation was incubated in the buffer solution of the low [Ca^2+^] for 5–9 min in advance, then the low [Ca^2+^] incubation was started by adding water. After incubation for 37 min, tightly elongated vesicles still had comparable two-rail or tetragonal crystalline arrays (Figure 8a–c). After a 93-min incubation, crystal arrays in the elongated vesicles were undoubtedly degraded (Figure 8d–f). Orderly arrays were no longer found after overnight incubation (Figure 8g,h). In contrast to the assumption mentioned above, these results suggest that prolonged incubation of the scallop SR preparation in low [Ca^2+^] without ATP degrades their crystal arrays.

**Figure 9:**
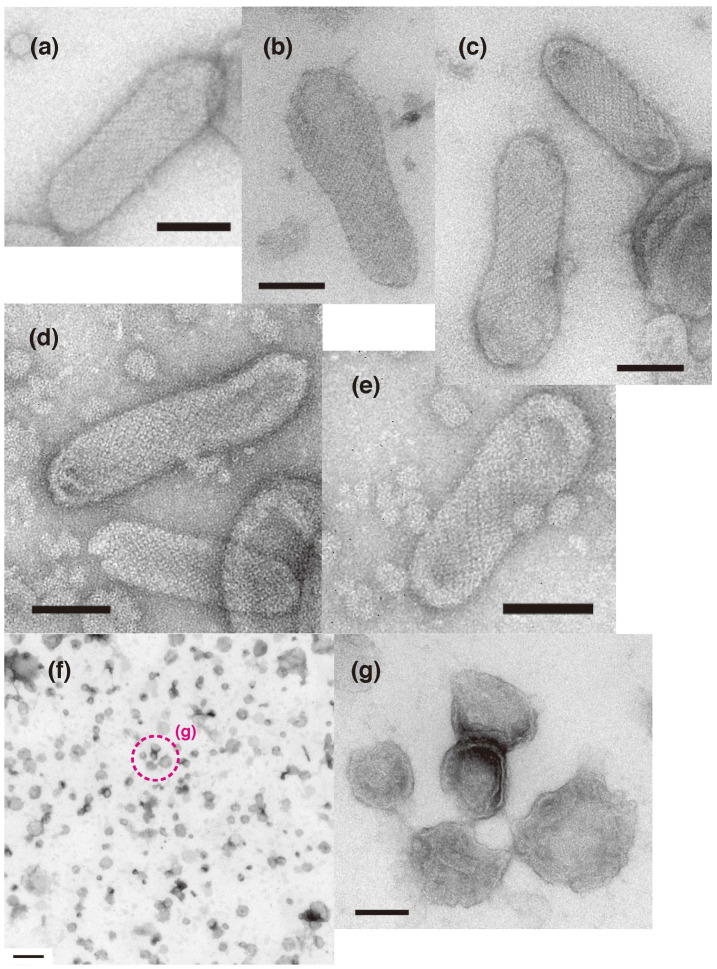
Time course of the morphology of SR vesicles during incubation at a low calcium concentration (~0.002 µM) with 5 mM ATP. (**a**–**c**) SR vesicles after 1 min. (**d**,**e**) SR vesicles after about 2.5 h. Because all ATP present was consumed during the 2.5 h incubation (see main text), “two-rail” arrays degraded, and tetragon arrays dominated. In (**a**–**e**), before the addition of ATP, the SR preparation was preincubated at a low [Ca^2+^] (~0.002 µM) for 5–9 min (see “Section 4. Materials and Methods”). (**f**,**g**) SR vesicles reacted with ATP for 1 min after overnight incubation at the low [Ca^2+^] in the absence of ATP. Panel (**g**) is a higher magnification image of the dotted circle (**g**) in panel (**f**). Crystalline arrays were not observed. Scale bar in (**a**–**e**,**g**): 100 nm. Scale bar in panel (**f**) 0.5 µm.

The time course of crystal formation was next examined at a low [Ca^2+^] (~0.002 µM) in the presence of ATP (Figure 9). After the SR preparation had been pre-incubated for 5–9 min without ATP and incubated for 1 min with 5 mM ATP, “two-rail” arrays of high crystallinity appeared (Figure 9a–c). Their features seem to be very close to those of arrays observed earlier [7,8,19], which were comprised of scallop Ca^2+^-ATPase molecules [20]. On the other hand, the scallop SR preparations observed earlier and here have, respectively, high (~85%) [7] and low (40–50%) [15]) Ca^2+^-ATPase purities relative to the total amount of SR proteins present. Together, these results indicate that the highly regular ATPase crystalline arrays were comprised of ATPase. The low ATPase purity of our SR preparation is attributable to contaminating other proteins and muscle membrane-fragments other than SR (see in [15]). By contrast, the distinct ATPase tetramer arrays with an area of almost 100 Å, observed in the absence of ATP (Figure 3b,b’) disappeared after the addition of ATP. The highly ordered “two-rail” arrays observed after 1 min incubation at a low [Ca^2+^] (~0.002 µM) with ATP were also observed in the tightly elongated vesicles after 16 min incubation with the low [Ca^2+^] (~0.002 µM) and ATP (data not shown). After 2.5 h incubation with ATP, “two-rail” arrays degraded, instead tetragon arrays dominated (Figure 9d,e). The scallop SR preparation has been found to hydrolyze about 1.8% (~0.09 mM) of the added ATP (5 mM) in 10 min under the condition of 0.05 mg SR protein/mL at a low [Ca^2+^] of ~0.01 µM [15]. In the present study, the SR vesicles contained 0.3 mg SR proteins/mL, which is 6 times higher than the vesicle protein concentration employed in the earlier study [15]. Based on the above-mentioned parameters describing the ATP hydrolysis, it is approximately calculated that the SR vesicle preparation consumed almost 10% (~0.54 mM) of the added 5 mM ATP in 10 min. Namely, the preparation hydrolyzed 3.2 mM ATP (64% of the added ATP) in 1 h. It is, therefore, probable that the ATP in the reaction mixture was almost completely consumed by the SR vesicles during the prolonged incubation time of about 2.5 h.

During the reaction, ATP is converted to ADP. ADP has been shown to stabilize the structure of detergent-solubilized rabbit Ca^2+^-ATPase of the rabbit SR [21]. However, ADP has also been found to inhibit ATP-supported calcium transport by SR [1]. It is, therefore, considered that ADP is not substitutable for ATP to maintain the physiological function of the ATPase protein. On the other hand, after SR vesicles were incubated at ~0.003 µM Ca^2+^ without ATP overnight, addition of ATP did not improve the crystallinity of the ATPase arrays in vesicles (Figure 9f,g). The instability of tetragonal crystalline array disposition at this low calcium concentration without ATP, may be associated with the instability of SR Ca^2+^-ATPase activity in a similar environment reported earlier by Kalabokis et al. [22]. The effect of ATP on Ca^2+^-ATPase crystallization was precisely analyzed at various [Ca^2+^] in the second part of our study.

The tubular vesicles displaying tetragon, two-rail and monomer Ca^2+^-ATPase arrays, observed here and in the second part of our study, generally had a diameter of about 100 nm, but the diameters of the actual arrays present varied. The diameter of “two-rail arrays” varied least; the diameter of tetramer arrays varied less than the diameter of monomer arrays. However, the crystallinity of the ATPase within most of the tubular vesicles was generally not very good. The difference seems to be related to rather straight shapes of cylinders of “two-rail array”.

### 2.2. Thapsigargin Inhibits ATPase from Crystallization and Restrains SR Vesicles from Elongation

**Figure 10:**
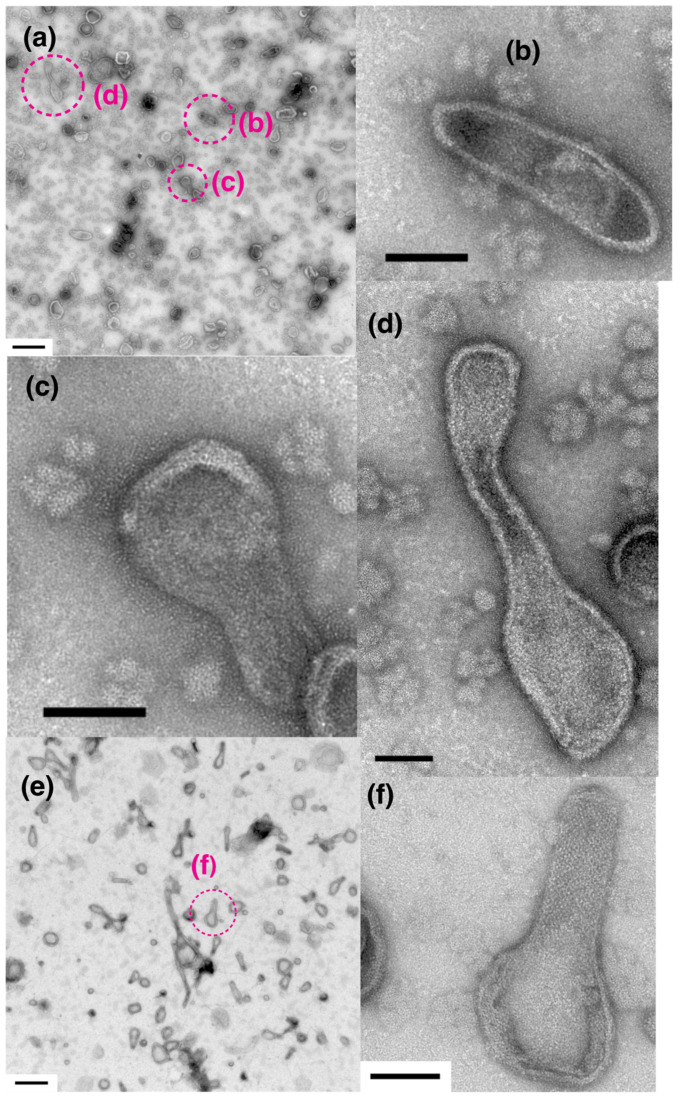
Effect of thapsigargin (TG) on the crystallization of 40 Å ATPase particles of SR vesicles in the presence of ATP. (**a**–**d**) SR vesicles with TG (2 µM) and dimethylsulfoxide (DMSO) (0.12% (*v*/*v*)). (**b**–**d**) are higher magnification images of the dotted circles (**b**–**d**) in (**a**), respectively. (**e**,**f**) Vesicles treated with DMSO alone. (**f**) Higher magnification image of the dotted circle (**f**) in panel (**e**). In (**a**–**d**), TG was added to the reaction mixture at the ratio of 6.7 nmol/mg SR protein, assuming that 40–50% of the total protein present was Ca^2+^-ATPase [15]. The ratio of TG to the scallop Ca^2+^-ATPase protein (13.4–16.8 nmol TG/mg of the ATPase protein) was higher than that (10 mmol/mg Ca^2+^-ATPase protein of the rabbit SR) [23] required for the complete inhibition of the Ca^2+^-ATPase activity of the Ca^2+^-ATPase rich (~90%) rabbit SR. After the TG treatment, the SR was reacted with ATP for 1 min. Scale bar in panels (**a**,**e**): 0.5 µm. Scale bar in panels (**b**–**e**): 100 nm.

To understand the effect of thapsigargin (TG) on Ca^2+^-ATPase crystallization, SR vesicles were next treated with TG (2 µM) and dimethylsulfoxide (DMSO) (0.12% (*v*/*v*)) before addition of ATP (see Methods and Figure 10 legend for details). In the presence of TG, crystalline arrays were neither observed in tightly elongated vesicles (Figure 10a–c) nor in crookedly elongated vesicles (Figure 10a,d). This agrees well with the notion that the 40 Å surface particles are Ca^2+^-ATPase because TG specifically reacts with rabbit ATPase to form a catalytically inactive dead-end complex [23]. As a control experiment, SR vesicles were incubated with DMSO alone, because DMSO was used as a solvent for TG. In this control, crystalline array formation was confirmed (Figure 10e,f), although the crystallinity of the observed arrays might be lower than the crystallinity of arrays formed without DMSO (compare Figure 10f with Figure 9a–c), suggesting that DMSO may partially disturb crystallization of scallop Ca^2+^-ATPase.

## 3. Discussion

**Figure 11:**
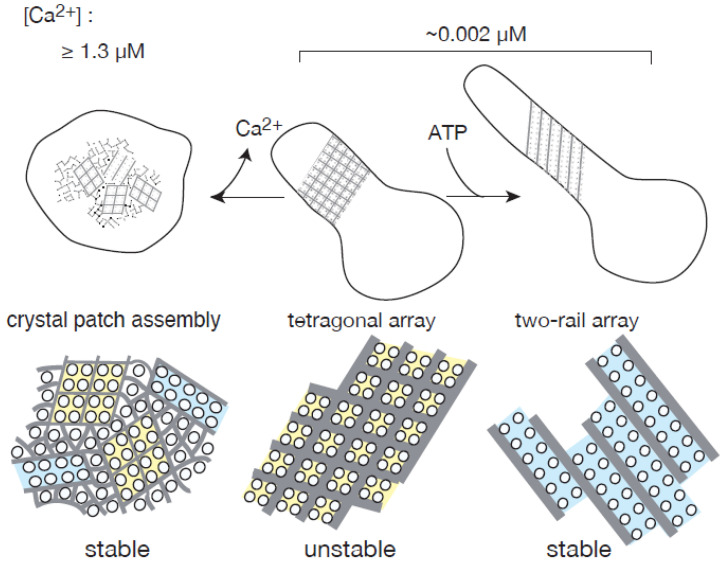
Schematic representation of the formation and development of a crystalline array of Ca^2+^-ATPase molecules in scallop SR. ATP stabilizes the vesicle’s crystalline array to elongate the vesicle.

The crystal patch, tetragon, “two-rail” and monomer dispositions of scallop ATPase molecules observed here (Figure 1, Figure 2, Figure 3, Figure 4, Figure 5, Figure 6, Figure 7, Figure 8, Figure 9 and Figure 10) are shown schematically in Figure 11. The data reported in the present paper are based on in vitro observations of the SR, which allowed a simplified system to be studied. They were performed using isolated scallop SR vesicles under the artificial, physiological conditions. The negative staining method employed has limitations because it involves staining with uranyl acetate and drying the sample [6]; some salt patterns might be formed during drying process and the drying process could itself disturb SR structures. Although these factors might have made the SR classification more complex, we consider the general trends reported to be representative and hope that the theory proposed will stimulate future in vivo study of the SR.

Ca^2+^ and ATP dependency of the four types of crystallization reported in this paper can be summarized as follows: (i) The crystal patch assemblies of ATPase molecules formed in the round vesicles stored at a high [Ca^2+^] (>180 µM) without ATP, presumably began to change into assemblies of ~100 Å square tetragons when the vesicles were incubated at a low [Ca^2+^] (≤1.3 µM) without ATP, and developed into tetragonal arrays (Table 1, Figure 3a–c). (ii) The tetragonal arrays were unstable at a low [Ca^2+^] without ATP (Figure 8d–h and Figure 9f,g), and (iii) ATP transformed the tetragonal arrays into more stable “two-rail” arrays of high regularity to elongate the SR vesicles (Figure 9a–c) like the intact SR tube [8] present in in myocytes in vivo. The monomer array dispositions of ATPase (Figure 3d,d’) are not considered in detail here, because the data about them are insufficient. They will be the subject of a future study. Stabilization of the crystalline arrays by ATP, can be described based on the biological hydrotrope model of ATP [24], like rabbit Ca^2+^-ATPase crystals [6], i.e., ATP may return the structure of the scallop Ca^2+^-ATPase molecules into their intrinsic state to help crystallization. The inhibition of ATPase “two-rail” crystallization by TG alone might be specific to scallop, because the formation of two-rail-crystalline arrays of rabbit Ca^2+^-ATPase promoted by decavanadate [25] (an inhibitor of Ca^2+^-ATPase [26]) was further enhanced by TG [23,27].

The scallop Ca^2+^-ATPase molecule is proposed to have a secondary structure comprised of ten transmembrane and five ‘stalk’ domains with two large cytoplasmic regions [28] like the rabbit Ca^2+^-ATPase [2,3]. In the presence of ATP, scallop Ca^2+^-ATPase molecules may also act as a membrane endoskeleton of the SR, elongating the SR, as proposed for rabbit SR Ca^2+^-ATPase [6]. The three-dimensional structure of the scallop “two-rail” array has been characterized as dimer ribbons of the Ca^2+^-ATPase molecules running diagonally around the tubular membrane of the isolated rod-shaped SR vesicle [19]. On the other hand, in an earlier observation of intact scallop striated muscle in the resting state, not only the cytoplasmic domain of the Ca^2+^-ATPase, but also its transmembrane domain, exhibit a crystalline arrangement and have close contact with each other in the elongated SR tubes [8]. These earlier observations agree with the above proposal that the Ca^2+^-ATPase molecules may act as a membrane endoskeleton serving to elongate the SR. In the second part of this study, the stable scallop SR preparation employed here was used to precisely analyze the stabilization of ATPase crystals by ATP at various Ca^2+^ concentrations to deduce the physiological role of ATPase membrane endoskeleton in vivo formed and will be reported shortly.

## 4. Materials and Methods

Scallop SR was isolated from the cross-striated adductor muscle of the scallop (*Patinopecten yessoensis*) [15], and to minimize damage to SR during homogenization of the muscle tissue, NaHCO_3_/HCl buffer, which is generally non-toxic for cells, was used. The isolation procedure was as follows (see in [15] for details). Active scallops were selected by keeping them in aerated seawater overnight at 4–12 °C; scallops, extending their vela near the rim of the shell, were used. The striated portion of the adductor muscle was rinsed with 0.7% NaCl (weight/volume) and cut into 5–10 mm cubes with stainless steel scissors. One-hundred grams of these muscle cubes was homogenized with 500 mL of 50 mM NaHCO_3_/HCl (pH 7.0) by using a Waring Blender at maximum speed (18,000 rpm) for 15 s 4 times. The homogenate was centrifuged for 20 min at 1500× *g* to remove myofibrils and muscle tissue which was not fully homogenized. The supernatant was centrifuged for 50 min at 10,800× *g*. The obtained supernatant was further centrifuged for 50 min at 40,000× *g* to sediment the SR fraction. The pellet in the centrifuge tube consisted of a dense and viscous, white and partially transparent lower portion with a soft and easily dispersible, milk-white upper portion. One to 3 mL of 0.6 M KCl in 20 mM Tris/maleate (pH 7.0) was added to each of the tubes. The soft, upper portion of the pellets slid off the dense lower portion when the tube was gently shaken. This upper portion was transferred to a Dounce homogenizer and homogenized using a Teflon pestle. The volume of the homogenized suspension was increased to about 30 times that of the soft pellets by addition of 0.6 M KCl solution containing 20 mM Tris/maleate (pH 7.0) and allowed to stand for about 30 min to permit solubilization of the contaminating actomyosin. After that, the suspension was centrifuged for 45 min at 55,000× *g* to separate the SR fraction from the solubilized actomyosin. The pellet was homogenized in 0.12 M KCl containing 20 mM Tris/maleate (pH 7.0) and recentrifuged for 50 min at 45,000× *g*. The obtained pellet was suspended in the 0.12 M KCl solution mentioned above. This suspension containing SR fragments was stored as scallop SR preparation at −80 °C with ~0.3 M sucrose. It has been predicted that the amino acid sequence of the scallop SR Ca^2+^-ATPase [28] has ~70% overall similarity to that [2] of the SR ATPase from the adult fast-twitch skeletal muscle of rabbit, and that one type of the ATPase gene is expressed in the scallop striated muscle cells. It has been shown that the SR preparation has a high activity of Ca^2+^-transport, which is comparable with that of the rabbit SR preparation [14,15].

For electron microscopic study of the negatively stained SR, the SR (0.3 mg of protein/mL) was incubated with 100 mM imidazole buffer (pH 7.0) containing 0.12 KCl, 5 mM MgCl_2_, ~0.002–69 µM Ca^2+^ with and without 5 mM ATP at 12 °C for 1 min, unless otherwise indicated; before the addition of ATP or water (as a control without ATP), the SR was stood in the buffer solution for 5–9 min. Calcium concentration was calculated with the help of calcium buffer using EGTA, taking into account of the total calcium, ionic strength and pH in the SR-containing buffer solution with and without ATP, reported earlier [29,30,31]; in the calcium buffer, a low concentration (~0.002–10 µM) of calcium is stabilized in the buffer solution containing higher concentrations of calcium (~26–566 µM) and EGTA (0.5–5.0 mM). The calibration of microelectrodes and fluorescent probes for [Ca^2+^] had been performed in the calcium buffer solution. The total calcium was composed of the added (10–550 µM) and contaminated (16 ± 7 µM (n = 4)) calcium; the contaminated calcium was measured by the method of atomic absorption. The association constants for EGTA-calcium with and without ATP were taken as 1.9539 × 10^6^ and 2083 × 10^6^ M^−1^, respectively [32]. In the presence of ATP, the formation of ATP-calcium and ATP-magnesium was considered; the association constants of ATP-calcium and ATP-magnesium were taken, respectively, as 6.969 × 10^3^ and 1.239 × 10^4^ M^−1^ [33]. One drop of the SR suspension was placed for 30 s on a carbon-coated grid, followed by staining, two times, with a drop of 2% uranyl acetate. This procedure was carried out within a cooling box at 5 °C. The specimens were viewed with a JEM-1230 transmission electron microscope (JEOL, Tokyo, Japan) at 100 kV accelerating voltage.

## 5. Conclusions

The data obtained in the present paper suggest that (i) in the absence of ATP, low Ca^2+^ concentration (<1.3 µM) transforms tightly assembled crystal patches of Ca^2+^-ATPase molecules into “two-rail” or tetragonal spiral arrays in isolated round scallop SR vesicles prepared at a high Ca^2+^ concentration, and that elongation of the spiral arrays transforms some of the vesicles into tightly elongated vesicles. (ii) At a low Ca^2+^ concentration of ~0.002 µM, ATP increases the stability and regularity of the “two-rail” arrays increasing their appearance frequency, and this is accompanied by the disappearance of distinct tetramer-arrays. (iii) Thapsigargin (a specific inhibitor of the Ca^2+^-ATPase) prevents both the crystallization of ATPase molecules and the elongation of vesicles in the presence of ATP. (iv) In both round and tightly elongated vesicles, Ca^2+^-ATPase can form crystals: tightly assembled crystal patches and/or crystallized cylinders. Taken together, these results suggest that crystal structures formed by membrane proteins, membrane endoskeletons, might have physiological roles in the formation of the microscale structures of cells.

## Figures and Tables

**Table 1:** Calcium dependence of SR vesicle shape and disposition of the Ca^2+^-ATPase particles in the absence of ATP. The vesicles were classified, and the number of each type of the vesicle was counted at ~0.003–69.0 µM Ca^2+^ (see text for details); details of the classification are given in Table S1. First, all the vesicles (major axis > 0.065 µm) were classified as either elongated (ratio of the major axes to the minor axes ≥ 2) or round (ratio of the major axes to the minor axes < 2), then the elongated vesicles were sub-classified as tightly or crookedly elongated. The disposition states of their surface particles were classified as three types: ‘crystalline’, ‘tight assembly of crystal patches’ and ‘unclear’. The vesicle populations (46–222 vesicles) within the 5.4 µm by 5.4 µm electro-microscopic views, were subjected to the vesicle classification (see text for details); 2–4 views were analyzed at each calcium concentration. At 0.003–0.54 µM Ca^2+^, more than five tightly elongated vesicles were present in many views. At 1.3–19 µM Ca^2+^, only 1–3 elongated vesicles with and without crystalline array were observed in four views. At ~69 µM of Ca^2+^, no tightly elongated vesicle was observed, and two views did not include any tightly elongated vesicles. At ~69 µM Ca^2+^, less elongated vesicles were observed, and four views did not include any tightly elongated vesicles. Numbers in parentheses show the population percentages of each vesicular type relative to the total number of vesicles at the respective calcium concentrations. The full analysis for crookedly elongated and round vesicles is reported in Table S1.

			Calcium Concentration (µM)						
			0.003	0.03	0.11	0.54	1.3	19	69
elongated vesicles			47 (10.0%)	36 (6.1%)	9 (3.1%)	7 (1.7%)	12 (2.5%)	18 (4.2%)	1 (0.4%)
	tightly elongated		25 (5.3)	27 (4.6)	8 (2.8)	5 (1.2)	7 (1.5)	2 (0.5)	0
		with crystal-array	12 (2.6)	17 (2.9)	4 (1.4)	3 (0.7)	2 (0.4)	0	0
		without crystal-array	13 (2.8)	10 (1.7)	4 (1.4)	2 (0.5)	5 (1.0)	2 (0.5)	0
	crookedly elongated		22 (4.7)	9 (1.5)	1 (0.3)	2 (0.5)	5 (1.0)	17 (4.0)	1 (0.4)
round vesicles			421 (90.0)	557 (93.9)	279 (96.9)	394 (98.3)	465 (97.5)	407 (95.5)	284 (99.6)
total			486	593	288	401	477	426	285

## Supplementary Materials

The following supporting information can be downloaded at: https://www.mdpi.com/article/10.3390/ijms23063311/s1.

## Abbreviations

SR	sarcoplasmic reticulum
EGTA	ethylenebis (oxyethylenenitrilo) tetraacetic acid
TG	thapsigargin
DMSO	dimethylsulfoxide
TEM	Transmission electron microscopy/microscope
